# Therapeutic evaluation and analysis of complications of ethanol sclerotherapy for intramuscular vascular malformations: a single-center retrospective study

**DOI:** 10.3389/fsurg.2023.1274313

**Published:** 2023-10-24

**Authors:** Cai-Jun Jin, Qian Wang, Min Wang, Yong Chen, Si-Ming Yuan

**Affiliations:** ^1^ Department of Plastic Surgery, Jinling Hospital, Nanjing Medical University, Nanjing, China; ^2^ Department of Plastic Surgery, Jinling Hospital, Southern Medical University, Nanjing, China

**Keywords:** intramuscular venous malformations, ethanol, sclerotherapy, safety, efficacy

## Abstract

**Background:**

Intramuscular venous malformations (IMVMs) can cause pain and contracture deformity, leading to dysfunction of limbs. Ethanol sclerotherapy is one of the main treatments for IMVMs. This study aims to evaluate the efficacy and the complications associated with intravascular ethanol sclerotherapy for IMVMs and to provide a comprehensive summary of clinical experiences for future reference.

**Methods:**

A retrospective analysis was conducted on a cohort of 118 patients diagnosed with IMVMs who were treated with ethanol sclerotherapy in our center between 2006 and 2021. The plastic surgeons utilized a standardized collection *pro forma* to record the clinical data. Furthermore, a follow-up period ranging from 6 months to 5 years was implemented to assess the relief of symptoms, the change of lesion size, and the recovery of functional outcomes. In addition, an analysis of long-term complications was conducted.

**Results:**

The clinical symptoms of the patients in this group included pain, swelling, and limited movement. On average, 5.61 mL (range 2–14 mL) of ethanol was used during the sclerotherapy procedure. The intraoperative and early postoperative complications were successfully relieved by means of timely intervention, as observed during the follow-up period. Based on the MRI results, the sizes of the lesions in 19% of the cases were significantly decreased, while a slight decrease was observed in 39% of the cases. During the follow-up period, it was found that only eight out of the 118 patients included in this study experienced long-term complications related to sclerotherapy.

**Conclusions:**

Although ethanol sclerotherapy has proven to be an effective first-line treatment for IMVMs, it is associated with a variety of adverse reactions and short- and long-term complications. Surgeons are required to perform operations prudently and provide timely medical intervention for postoperative complications.

## Introduction

As the most common congenital vascular anomalies, venous malformations (VMs) could occur in many tissues or organs, including the skin and muscles, with approximately 40% of cases occurring in skeletal muscles. At birth, patients with intramuscular venous malformations (IMVMs) typically exhibit few symptoms and indications. As the patients grow, or encounter trauma, puberty, pregnancy, or other factors, the lesion can progressively enlarge, resulting in worsening of symptoms, such as pain, swelling, and even motor limitation. Therefore, timely treatment is needed for IMVM patients to alleviate the symptoms and control the size of the lesions. At present, there are various therapies for the management of IMVMs, including conservative treatment, sclerotherapy, operations, laser treatment, and cryoablation, among which sclerotherapy is the most commonly used. There are also many kinds of sclerosants available. Ethanol is the most widely used sclerosant in our center due to its high efficacy and potent destructive properties. Meanwhile, some scholars expressed that future studies should be directed toward investigating the anatomic location and the depth of the malformations. Unfortunately, there is a lack of systematic research pertaining to the utilization of ethanol sclerotherapy for IMVM. In this study, a retrospective analysis was conducted to evaluate the therapeutic effectiveness and complications associated with ethanol sclerotherapy in IMVMs.

## Methods

### Study design and patients

This study was reviewed and approved by the Medical Ethics Committee of Jinling Hospital. The study complies with the Declaration of Helsinki. Informed consent for the treatment and the publication of images was obtained from all the patients. The participants included in this study were individuals diagnosed with IMVMs who were treated with ethanol sclerotherapy in our center between January 2006 and December 2021. The sample consisted of 77 female and 41 male patients, aged 4–59 years with an average age of 23.17.

The inclusion criteria are as follows: (1) patients diagnosed with IMVM by MRI; (2) patients with signs or symptoms; (3) patients who had undergone ethanol sclerotherapy; and (4) patients with pre- and post-treatment data, followed up for more than 6 months. The exclusion criteria are as follows: (1) digital subtraction angiography or MRI revealing arteriovenous malformations, capillary malformations, lymphatic malformations, or combined vascular malformations; (2) patients who received foam sclerotherapy, intralesional copper wire retention or surgical resection and other treatments; and (3) patients with incomplete clinical data.

### Treatment

Following admission, all patients underwent routine examination to exclude surgery contraindications. General intravenous anesthesia or local infiltration anesthesia was administered according to the pain tolerance of the patients. Prior to the sclerotherapy procedure, all patients were administered 0.3 mg/kg of methylprednisolone intravenously to reduce the risk of complications such as cough and chest tightness. Based on the findings of the preoperative MRI and further imaging examinations, the lesion’s precise location was identified and subsequently accessed through percutaneous puncture using a 22-gauge scalp needle attached to a 10-mL syringe. The accuracy of needle positioning in the venous pouch was ascertained based on the venous blood reflux without obvious arterial pulsation through the needle. Some small lesions were punctured under ultrasound assistance. The treatment consisted of multifocal and repeated injections. The total amount of ethanol used in each treatment did not exceed 0.2 mL/kg. Throughout the injection process, vital signs and blood supply to the skin around the injection point were closely monitored. The treatment procedure was repeated after 2–3 months. Following the third treatment, an MRI scan was requested to evaluate the therapeutic effectiveness of the intervention and inform the determination of the subsequent treatment plan.

### Outcome assessment and follow-up

The patients in this group were followed up for a period of 6 months to 5 years, with an average of 41 months. We followed up with patients at the outpatient clinic. Those who could not be followed up through personal visits at the clinic were evaluated by a validated phone questionnaire. The details of the follow-up included the therapeutic effects of absolute ethanol sclerotherapy on relieving clinical symptoms such as pain and swelling. To assess the outcome in a more scientific and objective manner, the degree of symptom relief was divided into three categories: improvement, no change, and deterioration.

At admission, all the patients underwent an MRI examination. For patients with several treatments, an MRI examination was performed after three consecutive treatment sessions. The patients who received fewer than three treatment sessions would be scheduled for an MRI examination prior to their last follow-up. The changes in the size of the lesion were evaluated by measuring the maximum diameter observed using MRI before and after treatment with the following categorization: (1) no significant changes if less than 10%; (2) moderate reduction between 10% and 50%; and (3) marked reduction if higher than 50%.

### Complication analysis

After a sclerotherapy treatment, vital signs and blood supply to the skin around the injection point were closely monitored, and complications were recorded, such as blood supply disorder, ulceration, infection, nerve injury, superficial vein thrombosis, muscle contracture, and joint deformity.

Complications were categorized based on the timing of their occurrence: (1) intraoperative complications: complications that occurred immediately during the operation, including cough, chest tightness, and allergic reactions; (2) early postoperative complications: occurring within 1 month after sclerotherapy but could be cured by conservative treatment, including ulceration, infection, superficial vein embolization, and hematuria; (3) late postoperative complications: occurring beyond 1 month after sclerotherapy, including muscle contracture, joint deformity, and peripheral nerve injury.

### Statistical analysis

Statistical analysis was performed using IBM's SPSS Statistics 25.0 for Windows. The measurement data conforming to the normal distribution were expressed as mean ± standard deviation (*X* *± *SD), and a *t*-test was performed for comparisons between the two groups. Fisher's exact test was used for categorical data. Multiple groups were compared using the Kruskal–Wallis rank-sum test. A *P*-value < 0.05 was considered to indicate a statistically significant difference.

## Results

### Patient characteristics

A total of 118 patients with IMVM were enrolled in this study. IMVM lesions can be located all over the body, such as in the lower leg (*n* = 27), thigh (*n* = 32), upper limb (*n* = 22), trunk (*n* = 8), face (*n* = 11), and foot (*n* = 18). Meanwhile, 90 patients experienced pain, with 19 patients exhibiting symptoms of persistent pain, 39 patients reporting limited movement, and 62 patients presenting with swelling. The relevant demographic data, lesion location, symptoms, and imaging findings are shown in Table 1.

**Table 1:** Characteristics of patients.

	*N*	Percent (%)	Average	Range
Sex				
Male	41	35		
Female	77	65		
Age of presentation			28.53	12–56
Age of onset			18.84	11–42
Length of stay			1.89	1–5
Location of IMVM				
Calf	27	23		
Thigh	32	27		
Upper extremities	22	19		
Trunk	8	7		
Head and neck	11	9		
Foot	18	15		
Symptoms				
Pain	109	92		
Swelling	62	53		
Motion limitation	39	33		
Morphology of lesions				
Focal mass type	64	54		
Focal infiltration type	32	27		
Diffuse infiltration type	22	19		
Number of lesions				
1	77	65		
2	25	21		
>2	16	14		

All patients were routinely examined using MRI at the first admission to evaluate the range and location of the lesions. Based on the imaging findings, IMVM was categorized into three types: (1) Focal mass type (*n* = 64): the lesions were localized with well-defined margins. (2) Focal infiltration type (*n* = 32): the lesions were diffused in one muscle. (3) Diffuse infiltration type (*n* = 22): the lesions were extensive in more than one muscle with margins ill-defined.

There were no statistically significant differences observed in the levels of pain experienced by patients in each group. However, there was a statistically significant difference in swellings of patients not only between focal mass type and focal infiltration type (*P* = 0.001) but also between focal mass type and diffuse infiltration type (*P* ≈ 0.000), while there was no statistically significant difference observed between focal infiltration type and diffuse infiltration type (*P* > 0.05). The difference in motion limitation was statistically significant between not only focal mass type and focal infiltration type (*P* ≈ 0.000) but also focal mass type diffuse infiltration type (*P* = 0.000). Still, there was no statistically significant difference between focal infiltration type and diffuse infiltration type (*P* > 0.05).

### Sclerotherapy treatment

The cohort of 118 patients underwent a total of 378 treatment sessions (1–10 times per patient), with an average of 3.20 sessions per patient. In each session, 2–16 mL of absolute ethanol was injected, with an average of 5.61 mL and a maximum dosage of 0.2 mL/kg per patient.

### Outcome assessment and follow-up

**Figure 1:**
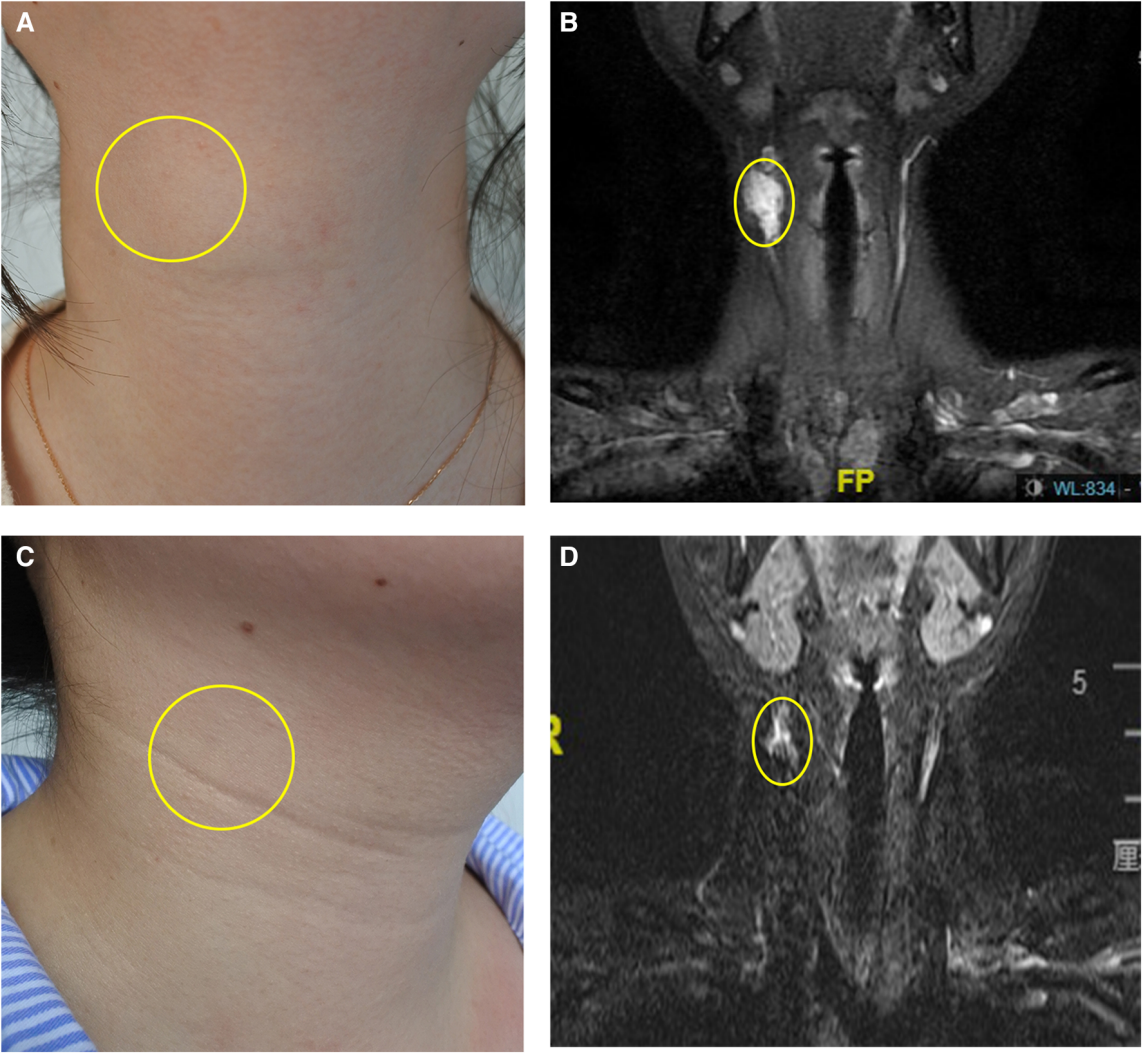
Focal mass type IMVM in the right neck. (**A**) The appearance before treatment. (**B**) The MRI before treatment. (**C**) The appearance after two times of treatment. (**D**) The MRI after treatment. The yellow ellipse indicated the lesion. The copyrights of the figure were held by corresponding author.

**Figure 2:**
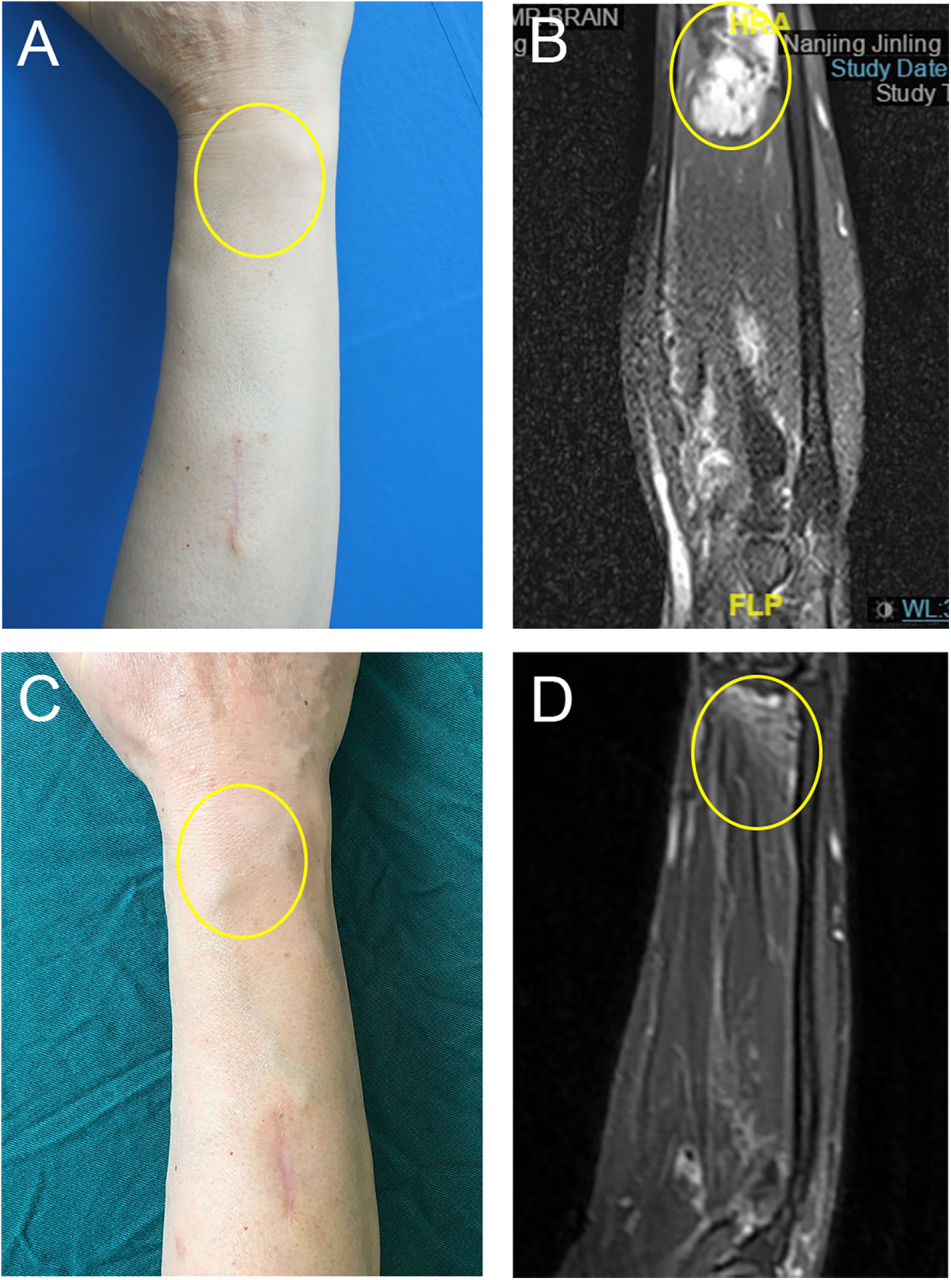
Focal mass type IMVM in the right upper extremity. (**A**) The appearance before treatment. (**B**) The MRI before treatment. (**C**) The appearance after three times of treatment. (**D**) The MRI after treatment. The yellow ellipse indicated the lesion. The copyrights of the figures were held by corresponding author.

**Figure 3:**
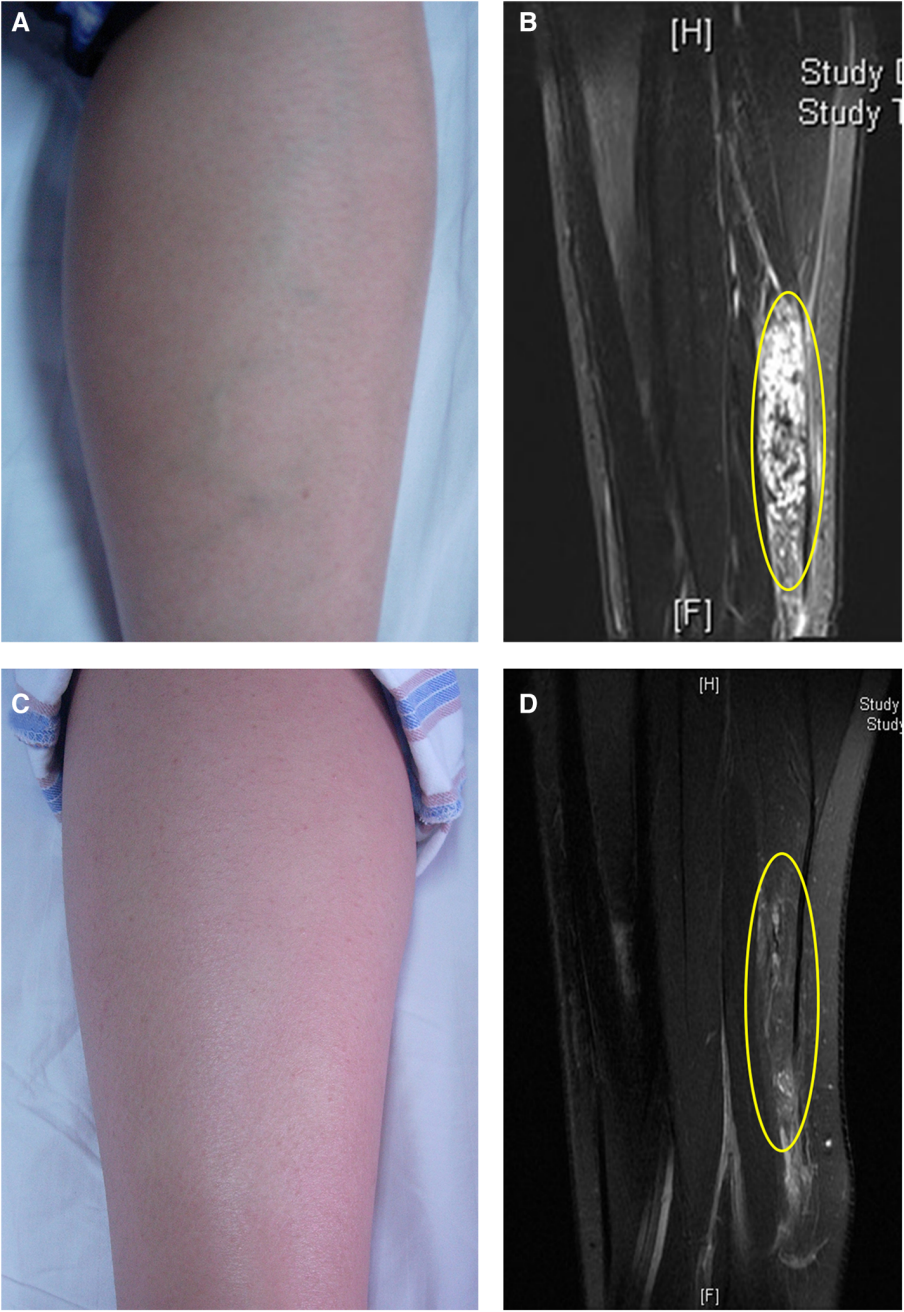
Focal mass type IMVM in the left calf. (**A**) The appearance before treatment. (**B**) The MRI before treatment. The yellow ellipse indicated that the lesion was located in the gastrocnemius muscle. (**C**) The appearance after three times of treatment. (**D**) The MRI after treatment. The yellow ellipse indicated the lesion. The copyrights of the figures were held by corresponding author.

The follow-up data of 118 patients are shown in Table 2. Among the 118 patients, 109 experienced pain, of which 63 (58%) cases had improved after ethanol sclerotherapy. A total of 62 patients were complicated with swelling, of which 34 (55%) cases had improved. A total of 39 patients experienced motion limitation, of which 17 (44%) cases had improved. Among them, there was a statistically significant difference in the improvement of pain among the three groups (*H* = 13.130, *P* = 0.001). In addition, there was a statistically significant difference in the swelling of patients not only between focal mass type and focal infiltration type (*P* = 0.015) but also between focal mass type and diffuse infiltration type (*P* = 0.009). Under MRI examinations, the lesions of 22 patients (19%) showed a remarkable reduction in size, as the typical cases shown in Figures 1–3. The lesions of 46 patients (39%) showed a moderate reduction in size, while that of 50 patients (42%) showed no significant change.

**Table 2:** Ethanol sclerotherapy and its outcomes.

	Focal mass type (*n* = 64)	Focal infiltration type (*n* = 32)	Diffuse infiltration type (*n* = 22)	All
Overall sessions	222	86	70	378
Sclerosing sessions per patients	3.47	2.69	3.18	3.2 (1–10)^a^
Does of sclerosants	4.68 (2–8)^a^	5.97 (4–12)^a^	7.54 (6–14)^a^	5.61 (2–14)^a^
Clinical results				
Pain^b^ (*n* = 109)	61	28	20	109
Improved	45	11	7	63
No change	14	17	12	43
Worsened	2	0	1	3
Swelling (*n* = 62)	22^c^	22	18	62
Improved	10	13	11	34
No change	12	9	7	28
Worsened	0	0	0	0
Motion limitation (*n* = 39)	7^c^	17	15	39
Improved	4	8	5	17
No change	2	8	8	18
Worsened	1	1	2	4
MRI results				
Marked reduction	14	4	4	22
Moderate reduction	26	13	7	46
No significant change	24	15	11	50

^a^ The average (minimum–maximum).

^b^ The distribution of grade information in the “pain” group is statistically different, *H* = 13.130, *P* = 0.001.

^c^ The data in this group are statistically different from that in the other two groups.

### Complication analysis

The incidence of complications related to sclerotherapy is shown in Table 3, including the following: (1) intraoperative complications: cough (47/118) and chest tightness (13/118); (2) early postoperative complications: skin ulceration (15/118), superficial vein embolization (2/118), hematuria (39/118), and infection (3/118); and (3) late postoperative complications: muscle contracture (5/118), joint deformity (2/118), and peripheral nerve injury (3/118). The above complications were relieved after symptomatic treatment. Life-threatening complications such as hemolysis, heart failure, pulmonary embolism, and allergic reactions were not observed.

**Table 3:** Complications.

	*n*	Percent (%)
Intraoperative complications	51	43
Cough	47	40
Chess tightness	13	11
Allergic reaction	0	0
Early postoperative complications	58	49
Skin ulceration	15	13
Vein embolism	2	2
Hematuria	39	33
Infection	3	3
Heart failure	0	0
Coagulation disorder	0	0
Late postoperative complications	8	7
Muscle contracture	5	4
Joint deformity	2	2
Peripheral nerve injury	3	3

## Discussion

IMVM is a congenital chronic refractory disease, and the lesions are mostly located in the muscles of limbs, showing progressive growth (1). Most patients experienced varying degrees of pain, swelling, or motion limitation. More critically, the presence of limb contracture deformity can significantly decline the patients’ quality of life. Although VM is a benign lesion, it has the characteristics of destructibility and invasiveness, leading to the lack of muscle coherence, reduced muscle contractility, and potential bone deformities.

Currently, the treatment of IMVM is controversial. Some scholars believe that surgical resection should be the primary therapy for focal mass type, by which the deformed venous lesions can be completely removed (2). However, due to IMVMs invasive nature and deep location within the muscles, it is challenging to entirely eliminate the lesions with surgical intervention. In addition, it may lead to serious postoperative complications such as bleeding and muscle and nerve injury. Sclerotherapy is the most commonly used treatment for IMVM. This treatment involves injecting sclerosing agents or chemicals into the malformed veins, contacting with the vascular endothelium, which will result in aseptic inflammation, thrombosis, and fibrosis (3). Compared with surgical resection, sclerotherapy alleviates the burden and shortens hospital stay. Therefore, sclerotherapy should be preferred for IMVM patients with focal or diffuse lesions.

A wide variety of sclerosants are available, such as absolute ethanol, monoethanolamine oleate, bleomycin, sodium tetradecyl sulfate (STS), and polidocanol (4). According to the form, they can be also classified into liquid sclerosants and foam sclerosants. Ethanol is considered to be the most effective and destructive liquid sclerosant for treating lesions (5). In contrast, foam sclerosants such as STS and polidocanol are less destructive for tissues and thus needs more treatment sessions. In this study, all the subjects were treated using ethanol. Out of all the patients studied, 109 patients experienced pain, of which 63 (58%) patients demonstrated improvement; 62 patients experienced swelling, of which 34 (55%) patients had improved; and 39 patients had combined limitation of motion, of which 17 (44%) patients had improved.

With regard to complications following ethanol sclerotherapy, some researchers have confirmed that 0.25 mL/kg of ethanol is considered to be the critical threshold that may cause adverse reactions (6). In addition, the locations of IMVM lesions are relatively deep, hence reducing the likelihood of skin necrosis following ethanol sclerotherapy (7). The injection dose was controlled to less than 0.2 mL/kg. Patients were required for follow-up visits every 3 months, and the subsequent treatments were mainly based on symptom improvement or imaging results. The appropriate dose of ethanol for each injection and the multiple injections at an interval of 3 months can effectively reduce the occurrence of complications and achieve optimal efficacy step by step. The common complications of ethanol sclerotherapy include intraoperative complaints of chest tightness and dyspnea. However, administering glucocorticoids prior to treatment can effectively relieve these symptoms (8). The complications were categorized based on the timing of their occurrence into three groups: intraoperative complications, early postoperative complications, and late postoperative complications. In our study, intraoperative complications occurred in 51 patients, including 47 cases of cough and 13 cases of chest tightness, of which nine patients experienced both cough and chest tightness. No allergic reactions were observed. The above patients probably developed microthrombi in the blood vessels during ethanol sclerotherapy, which refluxed into the pulmonary capillaries and triggered choking. However, it was relieved by preoperative administration of hydrogenated prednisone and a brief period of intraoperative rest.

**Figure 4:**
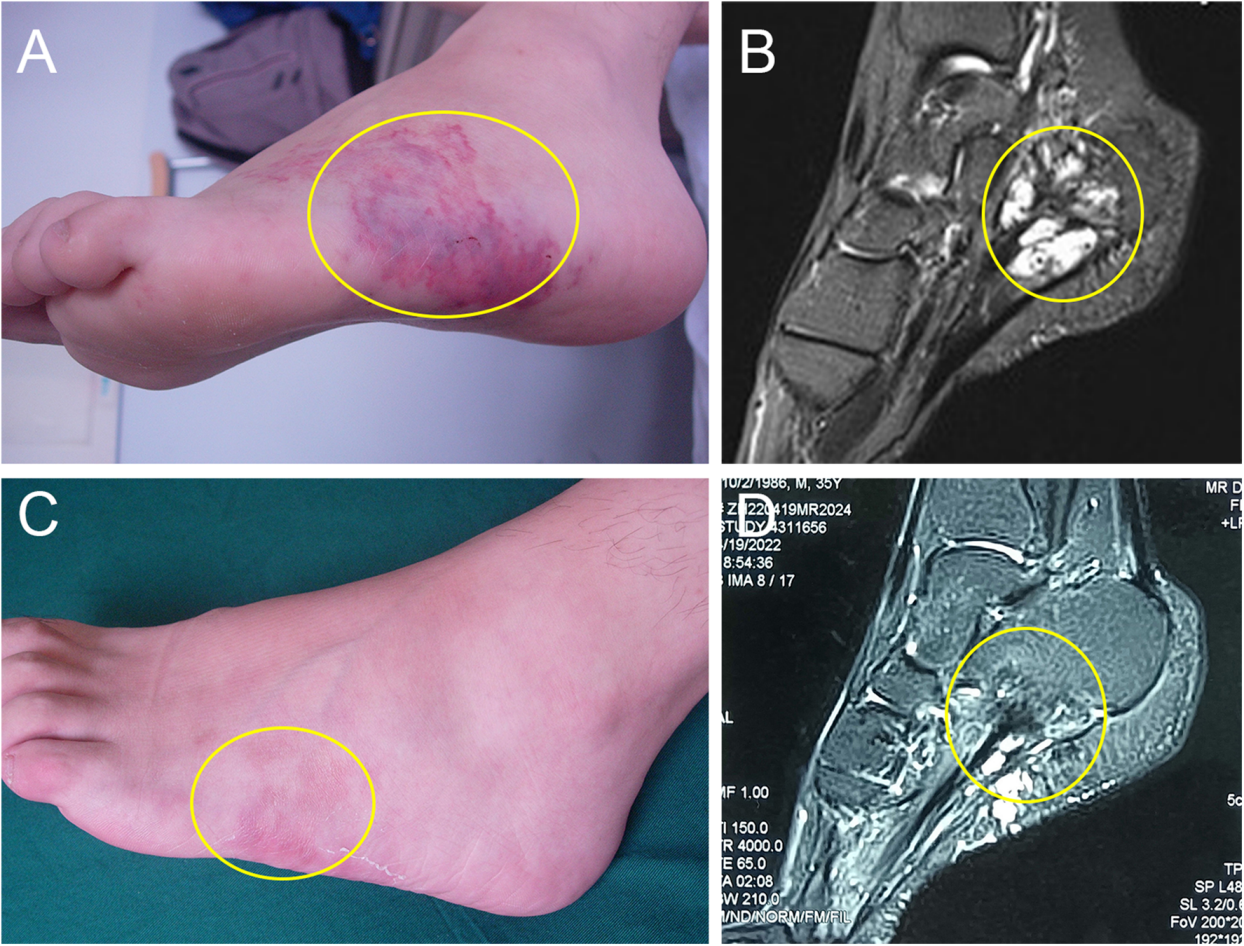
Diffuse infiltrative type IMVM in the left foot. (**A**) The immediate postoperative appearance. The local skin showed extensively damaged. (**B**) The MRI before treatment, which indicated that the lesions were located in the plantaris muscle. (**C**) The appearance after 4 weeks of treatment. The local skin was intact with no scarring. (**D**) The MRI after three times of treatment. The yellow ellipse indicated the lesion. The copyrights of the figures were held by corresponding author.

Petechiae and local skin necrosis observed in 15 patients were among the early postoperative complications in this study, of which 13 patients recovered completely after symptomatic treatment manifesting as improved microcirculation and enhanced venous return after regular dressing changes. Only two patients required a second operation, specifically skin grafting, to repair the secondary wound. The occurrence of localized skin petechiae and necrosis were caused by the destructive effects of ethanol on both the lesion and the return veins of the skin and subcutaneous tissue when destroying the deformity, which is a special phenomenon of sclerotherapy of IMVM. Among the two patients with venous embolism, one case was superficial vein embolism, which showed local skin petechiae and necrosis and was cured after regular dressing changes, as shown in Figure 4. The other case was a deep femoral vein embolism, characterized by swelling in the affected lower limb, the course of this condition lasted for more than 1 month, and the symptom gradually improved after anticoagulation treatment and wearing elastic stockings. Meanwhile, we observed that 39 patients had transient hematuria, all of which were hemoglobinuria. Some scholars believe that transient hematuria occurs after ethanol sclerotherapy due to the physiological reaction of the chemical nature of ethanol in the organism (9). We have not observed severe hemolytic gross hematuria requiring intravenous application of binding bead protein. As far as coagulation is concerned, our previous studies have found elevated fibrinogen degradation product and D–D in patients with ethanol sclerotherapy, and this elevation was often associated with a good therapeutic outcome; however, no serious disorders of coagulation were found (10).

Among the late postoperative complications, a total of eight patients were observed, and five of them experienced muscle contracture, which was relieved in three patients after regular stretching of the contracted muscles and the corresponding rehabilitation treatment. However, two patients refused to receive further treatment, resulting in a deformity of the corresponding joint. Muscle contracture occurs due to muscle fibrosis during ethanol sclerotherapy in IMVM, so patients should be treated at the early stage of contracture to prevent further fibrosis and joint deformity. If the muscle contracture is severe and the conservative treatment fails, surgical intervention may be considered as a viable option, including excision of the diseased muscle or a Z-lengthening of the Achilles tendon, the Hoke technique, and the Taylor Spatial Frame external fixation (11). Furthermore, three patients with peripheral nerve injury showed numbness in the innervated area. They were treated with neurotrophic therapy and completely recovered after 4–6 months.

To minimize the incidence rate of complications caused by ethanol sclerotherapy, surgeons should be extremely careful during the operation. Prior to the procedure, fully understanding the anatomy of the puncture site is necessary to avoid unwanted damage to the arteries and important nerves. During the procedure, confirming the puncture site could avoid leakage of sclerosant. If the lesion is too small to confirm the puncture in place, fluoroscopy or ultrasound could assist. After the procedures, it is essential to closely monitor the vital signs of the patient and conduct regular follow-ups for early detection and early intervention of postoperative complications. Meanwhile, in order to avoid damaging normal veins and ensure the accurate injection of anhydrous ethanol, it is necessary to perform fluoroscopy or ultrasound procedures due to the highly destructive nature of anhydrous ethanol. Particularly for VMs in the facial or cervical part and deep tissue, operation under the premise of visualization has been found to improve the accuracy of sclerotherapy and perhaps reduce the occurrence of complications to a certain extent, which could improve the efficacy of sclerotherapy.

To improve the effectiveness of ethanol sclerotherapy and reduce complications, researchers have developed ethanol gels. Because ethanol gel has a higher viscosity than liquid ethanol, it remains in the blood vessels for a longer period after injection into the vessels, resulting in a longer contact time with the lesion and a stronger effect on the local vessel wall. Thus, the ethanol dose and the occurrence of complications could be reduced, and the quality of life of the patients may be improved (12, 13). However, there are few clinical studies related to the use of ethanol gel, and additional research is required to confirm its effectiveness for clinical application in treating IMVM. Furthermore, some scholars reported simultaneous application of physical and chemical treatment could effectively destroy the endothelium of malformed veins (14, 15). In some huge and extensive infiltrative types of IMVMs, safe doses of sclerosants are often not enough for a good curative effect. Scholars tried to employ intravascular laser therapy for IMVM patients, in which the laser beams destroyed the lesion and the residual lesion was then treated with sclerotherapy. This kind of combined treatment also reduces the dose of sclerosants, thus decreasing the risks of complications or side effects.

## Conclusion

Within safe doses, ethanol sclerotherapy effectively shrinks lesions, reduces pain, relieves motion limitation, and improves appearance, with minimal occurrence of severe complications. Overall, ethanol sclerotherapy for IMVM is safe and effective and could be the preferred treatment for IMVMs. However, it is crucial for surgeons to perform operations prudently and provide timely treatment for postoperative complications due to the potential occurrence of multiple short- or long-term complications.

## Data Availability

The raw data supporting the conclusions of this article will be made available by the authors, without undue reservation.
